# Effect of the Consumption of Milk with Beta-Casein A2A2, Milk with Beta-Casein A1A2 and a Plant-Based Drink on Metabolic Health in Adults: Protocol IMPA-CT Study

**DOI:** 10.3390/nu17243922

**Published:** 2025-12-15

**Authors:** Jadwiga Hamulka, Magdalena Górnicka, Anna Berthold-Pluta, Adam Kalinowski, Marta Habanova, Dawid Madej

**Affiliations:** 1 Department of Human Nutrition, Institute of Human Nutrition Sciences, Warsaw University of Life Sciences (SGGW-WULS), 166 Nowoursynowska St., 02-787 Warsaw, Poland; jadwiga_hamulka@sggw.edu.pl (J.H.); dawid_madej@sggw.edu.pl (D.M.); 2 Department of Technology and Food Assessment, Division of Milk Technology, Institute of Food Sciences, Warsaw University of Life Sciences (SGGW-WULS), 166 Nowoursynowska St., 02-787 Warsaw, Poland; anna_berthold@sggw.edu.pl; 3 Health Centre (SGGW-Health Centre), Warsaw University of Life Sciences (SGGW-WULS), 166 Nowoursynowska St., 02-787 Warsaw, Poland; adam_kalinowski@sggw.edu.pl; 4 The Institute of Nutrition and Genomics, Slovak University of Agriculture in Nitra, Tr. A. Hlinku 2, 94976 Nitra, Slovakia; marta.habanova@uniag.sk

**Keywords:** milk A2, milk A1, plant-based drink, adults, dietary intervention, markers of metabolic health, bone turnover markers

## Abstract

**Background and Objectives:** Milk with A2/A2 β-casein (A2 milk) is currently the subject of numerous studies on the effects of its consumption on health. Commonly consumed milk contains a mixture of β-casein of different genetic variants (most often A1 and A2). In the polypeptide chain of A2/A2 β-casein, proline occurs at position 67, while in β-casein A1/A2, histidine occurs. The main goal of the dietary intervention was to identify and compare the effects of consuming A2 milk, conventional milk (A1) and oat drink on bone health, cardiometabolic health and immune system function in adults. **Methods:** The controlled IMPA-CT (Investigating Milk and Plant Alternatives Comparative Trial) Study was a randomized study with three groups (A2 Milk group, A1 Milk group, and Oat Drink group). The study included 162 adults with normal and/or overweight, without coexisting chronic diseases, aged 30–60 years. The intervention study consisted of the consumption of 500 mL of an appropriate product (A2 milk/A1 milk/oat drink) daily for 12 weeks. After qualification of the subjects, before the start of the study (T1′), in the 4th week of the study (T2′), in the 8th week of the study (T3′) and at the end of the study, after 12 weeks (T4’), an assessment of the diet and nutritional status was planned. Body composition, bone mineral density (DEXA) and biochemical tests were done. The primary outcome will be the effect of cow’s milk variants and oat drink consumption on bone health. Secondary outcomes will include changes in nutrient intake and cardiometabolic health as well as the immune system in adults. **Expected Results and Contributions:** The study design, including extensive follow-up and robust endpoint measures, contributed to understanding the therapeutic potential and safety profile or otherwise of β-casein A2/A2 milk and plant-based drinks.

## 1. Introduction

**Figure 1:**
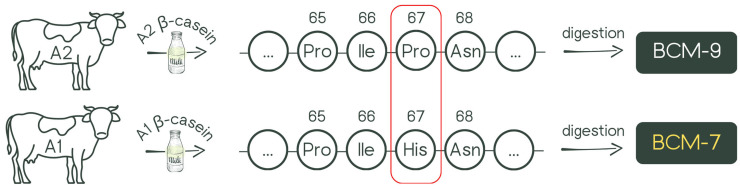
The difference in amino acids (His67/Pro67) between the A1/A2 and A2/A2 variants of β-casein and the release of β-casomorphins (BCMs).

Milk plays an important role in human nutrition, mainly due to its high nutritional value. It is a product that provides essential energy and nutrients in the early stages of mammalian life. However, while most mammals stop consuming milk after weaning, humans continue to consume it into adulthood [1]. Cow’s milk proteins, in particular caseins, are characterized by significant genetic diversity, comprising 13 variants: A1, A2, A3, B, C, D, E, F, G, H1/H2, I, and J. Variants A1 and A2 are the most common, and their key difference is the presence of histidine (His) at position 67 in variant A1 and proline (Pro) at the same position in variant A2 (Figure 1). This single amino acid change significantly impacts the digestion of β-casein by humans. In the case of β-casein A1, digestive enzymes and the gut microbiome break it down, releasing the heptapeptide β-casomorphin-7 (BCM-7) [2]. BCM-7 belongs to a group of peptides with opioid properties, commonly called casomorphins, whose formation has also been observed during milk processing or fermentation [3].

Current research indicates that BCM-7 may influence the regulation of the digestive system and blood pressure, but also has anticoagulant, antioxidant, and antimicrobial properties [4]. However, it is worth noting that BCM-7, as a potent opioid peptide, may also have adverse health effects through allergic reactions and prolonged milk passage through the digestive system, leading to intestinal inflammation [2,5,6]. Epidemiological studies also show a link between consuming milk containing β-casein A1 and an increased risk of developing type 1 diabetes and cardiovascular disease [7,8,9]. BCM-7 may interact with opioid receptors in the central nervous system and gastrointestinal tract, highlighting its potential impact on human health [3]. On the other hand, de Gaudry et al. [10] showed no significant association between A1 milk and cardiovascular disease, diabetes, or neurological diseases. Daniloski et al. [11] obtained identical results in clinical and animal studies. Therefore, the issue of BCM-7 formation and its impact on human health remains unresolved. The scientific literature emphasizes the need for further research to better understand the potential risks associated with consuming dairy products containing β-casein A1/A2. It is particularly important to note that adverse effects have been observed in animal studies, while clinical evidence from human studies is limited [1,12].

In response to these concerns, a new type of cow’s milk called “A2 milk” has appeared on the market and has gained popularity in many countries. Thanks to cow genotyping techniques and the selection of appropriate bulls and calves, it is possible to breed cattle that produce only A2 milk [3]. This type of milk is a product devoid of the β-casein A1 variant and contains β-casein A2/A2, which, due to its more difficult digestion, limits the release of BCM-7 [13]. It has been shown that the concentration of BCM-7 in the intestines is four times higher when produced from milk from A1/A2 cattle than from homozygous A2/A2 cattle [14]. Furthermore, BCM-7 can be detected in the blood after digestion of A1 milk but not after consumption of A2 milk [15]. It is also worth noting that human milk also contains mainly A2/A2 β-casein [3]. The introduction of A2 milk into the diet may, therefore, be a solution to some of the health problems associated with the consumption of traditional A1 milk. However, further research is needed to understand the impact of β-casein A2/A2 on human health as well as the potential mechanisms of action.

On the other hand, plant-based beverages have recently become popular as an alternative to cow’s milk. Derived from various plant materials, including cereals, legumes, nuts, and seeds, these products are naturally lactose-free and suitable for individuals with lactose intolerance or a cow’s milk protein allergy. Growing consumer awareness of the environmental impact of animal farming, ethical concerns, and interest in plant-based diets have further driven demand for these beverages. However, the nutritional value of plant-based beverages differs markedly from cow’s milk and is highly variable, depending on the type of raw material used and the production technology employed [16,17].

Although soy-based drinks are the only plant-based option with a comparable protein content to cow’s milk, most other plant-based beverages have lower protein levels and inconsistent amounts of essential vitamins and minerals. Therefore, many commercially available products are fortified with calcium, vitamin D, vitamin B12, and other micronutrients to resemble the nutritional profile of cow’s milk more closely [18,19,20]. Despite their growing popularity, the long-term health effects of replacing cow’s milk with plant-based beverages are insufficiently studied, particularly about bone health, metabolic parameters, and cardiovascular outcomes. Given this uncertainty and the variability in nutritional profiles, it is essential to evaluate the health effects of these beverages within controlled dietary interventions, particularly when they are used as regular milk substitutes in adult diets [18,19,20].

Given the need for further research on A2 milk and plant-based milk substitutes, this study was designed to evaluate their impact on adults by comparing the consumption of A2 milk, conventional milk (A1/A2), and cow’s milk substitutes. While recent study protocols such as the YUMMI Study [21] have addressed the effects of ruminant milk consumption on digestive comfort and nutritional status in older women, the scope, population and outcomes assessed in our IMPA-CT Study differ significantly. The YUMMI protocol primarily focuses on digestive tolerance and nutritional adequacy in a specific age group; in turn, our study evaluates a broader range of outcomes, including bone health, cardiometabolic parameters, immune function, and body composition, in a mixed adult population aged 30–60.

This dietary intervention aimed to identify and compare the effect of A2 milk, conventional milk, and oat drink on (i) bone health, (ii) cardiometabolic health, and (iii) the immune system in adults.

## 2. Materials and Methods

### 2.1. Ethics Approval

The study protocol was approved by the Rector’s Committee for Research Ethics Involving Human Participants at the Warsaw University of Life Sciences, on 29 November 2024, Poland (Resolution No. 38/RKE/2024). It was conducted in accordance with all procedures that adhered to the ethical standards for human research, as well as followed the Consolidated Standards of Reporting Trials (CONSORT) reporting guideline for trial studies. The study was explained to the participants before the start, and written informed consent was obtained from the participants.

### 2.2. Study Design and Participants

**Figure 2:**
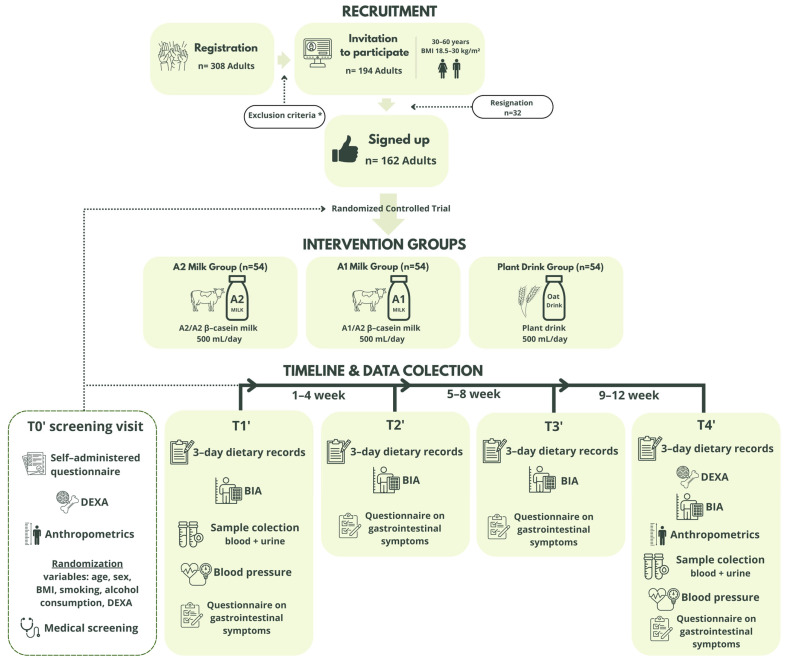
Flowchart: study design and sample collection (created by Canva). * Exclusion criteria: pregnancy or lactation; implanted medical devices (e.g., pacemaker, defibrillator); previous radiotherapy or chemotherapy; significantly modified diet; unable to provide informed consent; unable or unwilling to comply with study procedures; history of gastrointestinal surgery or disorders (e.g., IBD, ulcerative colitis, celiac disease, Crohn’s disease), cardiorespiratory problems, uncontrolled, diabetes mellitus, bleeding disorders.

The IMPA-CT study is a 12-week multi-arm, parallel, randomized trial conducted at the Warsaw University of Life Sciences, Poland. A total of 162 participants were randomized to one of three intervention groups: habitual diet supplemented with 500 mL of milk A2 daily for 12 weeks; or a habitual diet with 500 mL of milk A1/A2 daily for 12 weeks; or habitual diet supplemented with 500 mL of oat drink daily for 12 weeks to study the effects of long-term consumption of these types of drinks on bone density and their remodeling, metabolic health and nutrient intake among adults. Milks (A1 and A2) and fortified oat drink are widely available in our food market, and we acquired products from one of the largest dairy companies in Poland. Basic nutritional values are shown in Table 1. Milk and oat drinks were supplied by our research team. Participants consumed their assigned milk or oat drink, attended clinic visits for measurements and biological sample collection and completed validated questionnaires over the course of 12 weeks (Figure 2). At baseline, a subgroup of participants from each group had sequential blood and urine samples taken and completed assessments to evaluate their general metabolic health and bone remodeling in response to consuming the study drinks.

A total of 162 healthy adults were recruited from the general population by a combination of online and community-based recruitment strategies. All individuals interested in participating in the study were invited to a screening examination and assessed for eligibility according to the inclusion and exclusion criteria listed below.

The inclusion criteria were as follows:

- Aged 30 to 60 years;
- Body mass index > 18.5 or <30 kg/m^2^;
- No diagnosed chronic disease, i.e., diabetes, cancer, kidney disease;
- Not taking medications/dietary supplements that may affect carbohydrate and/or lipid metabolism.

The exclusion criteria as follows:

- Pregnancy or lactation in women;
- Implanted medical materials such as: pacemaker, defibrillator, stent, metal suture in the heart or blood vessel, and implants;
- Previous radiotherapy and/or chemotherapy;
- Significantly modified diet (e.g., ketogenic, vegetarian, or ovo-vegetarian) and health condition requiring a specialist diet;
- Unable to give informed consent;
- Unable or unwilling to comply with the study procedures;
- Have medical history of gastrointestinal surgery or disorders (inflammatory bowel disease, ulcerative colitis, coeliac disease, Crohn’s disease), cardiorespiratory problems, uncontrolled diabetes mellitus, bleeding disorders.

The non-exclusion criterion was the following:

- Hypertension, or depression that are well-controlled with medical intervention.

#### Screening, Blinding and Randomization

Respondents who expressed interest in the study completed an online pre-screening questionnaire to assess their current health status and lifestyle habits. This questionnaire consisted of questions covering the areas of contraindications, usual diet and physical activity. Volunteers were then invited to a screening visit to the Anthropometrics Laboratory, where they had the opportunity to ask questions about the study before providing informed consent. After this, a research assistant took basic body measurements (weight, height and waist circumference). Based on the results and answers provided, their eligibility for the study was determined. Respondents who qualified for the main study had their body composition measured (BIA, DEXA) and were referred to a laboratory for blood sampling.

Due to the nature of the intervention, which involved consuming commercially available products in their original packaging, neither the participants nor the research staff distributing the products could be blinded to the group allocation. However, to minimise detection bias, the laboratory personnel performing the biochemical analyses were strictly blinded to the participants’ group assignments. Anthropometric measurements and DEXA scans were conducted by research team members who were aware of the allocation. Nevertheless, DEXA and body composition analyses are automated, objective instrumental measurements; thus, the lack of assessor blinding had no impact on the accuracy or precision of the data generation.

Participants were randomly assigned to one of three groups: The A2 Milk Group, the A1 Milk Group and the Oat Drink Group, in a ratio of 1:1:1. The randomization sequence was computer-generated using STATISTICA (version 13.1 PL, StatSoft Inc., Kraków, Poland). Stratified block randomization was employed to ensure a balanced distribution of key prognostic factors across the groups. These factors included age, sex, BMI, smoking status, alcohol consumption and DEXA results (fat mass and BMD z-score) (see Table 2). Random permuted blocks of varying sizes (3 and 6) were used. Also, to ensure allocation concealment, the randomization list was kept by a researcher (D.M.) who was not involved in participant recruitment. Group assignment was revealed to the enrolment staff only after completing the baseline assessments.

### 2.3. Intervention

Once participants’ blood chemistry results were confirmed to meet study requirements, eligible individuals were formally enrolled and provided with links to appropriate questionnaires and surveys with instructions and the option to complete them online or on paper. Data was collected to assess their socioeconomic status and identify any important issues, which were used to classify participants into individual study groups (Table 2).

In the week prior to the entry visit (week 1), at weeks 4, 8, and the final week of the study (week 12), participants collected three-day dietary records on three randomly assigned days (2 weekdays, 1 weekend day). Participants underwent body composition assessment using bioimpedance analysis (BIA) at every check-up visit and dual-energy X-ray absorptiometry (DEXA) at the baseline and endpoint visits (see Figure 2).

To minimize variability in blood biomarkers due to dietary factors unrelated to the study intervention, participants were instructed on the timing and composition of meals consumed prior to blood collection.

During the study visits (T2′ and T3′), participants reported three-day dietary records on three randomly selected days (2 weekdays, 1 weekend day), and body composition was measured using the BIA method. Participants were provided with a supply of their assigned beverages every four weeks. They were asked to maintain their habitual diet and drink one serving of the beverage with breakfast and lunch twice daily (500 mL in total/d) for the 12-week intervention period. Each person taking part in the study was informed about the nutritional and energy value of the drink and instructed to follow the following recommendations:

- Do not change your usual diet: Do not make significant changes to your diet during the study to avoid affecting your results.
- Do not share milk with others: The milk you receive is intended solely for the study participants. Do not share it with others or feed it to animals.
- Consuming additional dairy/plant-based products: In addition to the designated amount of milk, you may limit other dairy products or dairy substitutes based on your usual intake.

### 2.4. Sample Collection

Before the baseline blood and urine samples were taken, each participant underwent a medical screening visit with a study physician. This included a physical examination and a review of the participant’s medical history, in order to exclude any contraindications to participation and confirm their general health status. Only those who passed this medical screening proceeded to the biochemical baseline assessments (T1′).

Blood samples were collected twice by qualified personnel in a medical laboratory—before the intervention (T1′, baseline) and after the intervention (T4′, final visit)—in a fasting state. On these same examination days, urine samples were also collected in the morning. All collected biological samples, both blood and urine from the baseline and final visits, were analyzed by the Medical Diagnostic Laboratory in Warsaw.

### 2.5. Monitoring and Compliance/Adherence

Strategies to reduce dropout included creating a supportive environment for participants, flexibility in scheduling laboratory visits (±2 days), sending reminders via email, and ensuring that participants received complete results. Study staff contacted participants in an agreed manner to discuss adherence and resolve any difficulties encountered. Support was offered in the form of counseling by a registered dietitian to help them improve adherence. To assess the consumption of the studied drinks, participants were asked to keep a food diary and during follow-up visits were asked about their self-assessment of compliance with the recommendations to drink 500 mL/d of milk or oat drink. Additionally, to further encourage participation, respondents received glycemic sensors for a period of 2 weeks. During these two weeks, detailed data on the consumption of the studied drinks and dietary intake were collected. 

### 2.6. Participation and Withdrawal from the Study

Participation in the study was voluntary, and participants had the right to withdraw from the study at any time. The principal investigator had the right to withdraw a participant from the study if they experienced significant adverse events, such as allergic reactions, deviation from the protocol, failure to comply with the study requirements, or withdrawal of consent.

### 2.7. Risks and Adverse Events

Although no significant adverse events had been reported for this type of study, and the study and intervention were low risk, participants might experience events such as gastrointestinal discomfort or discomfort due to intravenous access. Participants were informed of these risks before giving informed consent to the study. The research team monitored, recorded, assessed, and responded to adverse events. To evaluate these potential adverse effects, participants were asked to complete a short Questionnaire on Gastrointestinal Symptoms after the first study week (T1′), and again at each subsequent time point (T2′, T3′ and T4′). The questionnaire included questions regarding symptoms that could be experienced after consuming milk, such as diarrhea, bloating and other forms of gastrointestinal discomfort. In case of adverse events caused by the consumption of the milk or oat drinks during the intervention, the study physician was available to the participants and the event was recorded.

## 3. Study Outcomes and Measurements

### 3.1. Clinical Measurements

The participants’ height and weight were measured to calculate body mass index (BMI) as weight in kilograms per height in meters squared. Body height was measured by a stadiometer (Seca, Hamburg, Germany) and weight was measured by a digital scale (SECA 515mBCA, Hamburg, Germany).

Waist and hip circumference were measured by flexible nonelastic tape (Seca 203, Hamburg, Germany). These values were used to calculate the indices: waist–hip ratio (WHR) and waist-to-height ratio (WHtR).

All data were collected by trained researchers and measurements were taken according to the guidelines and protocols set out by NHANES [22]. All measurements were taken with shoes removed and in light clothing. All anthropometric measurements were taken in duplicate, with a third measurement taken should the first two differ by 10% and the average value was taken.

### 3.2. Body Composition

The analysis of body composition was performed with the bioelectrical impedance method (BIA) and dual-energy X-ray absorptiometry (DEXA; Lunar Prodigy, GE HealthCare, Chicago, IL, USA) according to the manufacturer’s protocols by a qualified person. The following measurement procedures were maintained for each participant: refraining from vigorous physical activity for at least 12 h before the test; no caffeine and alcohol consumption for 24 h before the test; avoiding certain medications such as diuretics, laxatives and electrolyte replacement drugs; fasting or 4 h after a meal; emptying the bladder 30 min before the test and removing any metal jewelry. Body Composition Analyzer InBody 770 (Inbody Co., Ltd., Seoul, Republic of Korea) with Lookin’Body 120, version 3.0.0.11 software, was used to estimate body fat content (%), fat mass (kg), free fat mass (kg), muscle mass (kg), total body water (kg, %), extra and intracellular water (%), visceral fat (in cm^2^), and phase angle (it correlates with metabolic status).

A DEXA scan using the Lunar Prodigy was performed to assess bone density and the risk of osteoporosis and fractures.

### 3.3. Blood Pressure

To assess the ankle–brachial index (ABI), blood pressure was measured at first and last visit (T1′ and T4′) in accordance with established guidelines and ESH/ESC recommendations. This process involved appropriate patient preparation, including at least 5 min of rest before measurement. A minimum of two readings were taken 1–2 min apart and their average calculated, with both the arm and ankle maintained at heart level. These measurements were conducted using an automated blood pressure system with correctly sized upper arm and ankle cuffs, while the patient was in a supine position.

### 3.4. Biochemical Analysis

During the baseline and final visits (T1′ and T4′), fasting blood samples and urine samples were collected to assess biomarkers of bone health, cardiometabolic health, and immune system functioning (Table 3).

#### 3.4.1. Biomarkers of Bone Remodeling (Bone Health)

Plasma calcium (Ca) and 1,25(OH)_2_D_3_ (calcitriol) were used as markers of bone homeostasis, and type I procollagen extension peptides (P1NP) and alkaline phosphatase (ALP) as markers of bone formation. Urinary calcium and deoxypyridinoline excretion, as well as plasma terminal cross-linked telopeptides of type 1 collagen (CTX1- for type 1 collagen), were determined as bone resorption markers. Because urine biomarkers were susceptible to potential changes in renal failure, to assess renal function, urinary creatinine excretion was determined [23]. Although urinary creatinine levels were not directly related to bone disease, they could indicate other diseases that might indirectly affect bone health. High creatinine levels, particularly in the blood, could suggest kidney problems, which could lead to calcium-to-phosphate disorders that negatively affect bones [24].

#### 3.4.2. Biomarkers of General Cardiometabolic Health and Immune System Functioning

The blood biochemical parameters of lipid profile (total cholesterol, triglycerides, high-density lipoprotein (HDL), low-density lipoprotein (LDL), creatinine, high-sensitivity *C*-reactive protein (hsCRP), uric acid, postprandial insulin markers—insulin, insulin-like growth factor (IGF)-1 and IGF-binding protein (BP)-3 (IGFBP-3), and postprandial glycemia were determined. As a casein allergy marker, sIgE was evaluated, and IgA and IgG were evaluated as markers of immune system functioning.

#### 3.4.3. Biochemical Characteristics of Study Participants After Randomization

Table 4 shows the baseline biochemical characteristics of the study participants by intervention group. At baseline (T1′), there were no statistically significant differences between the A2 milk, A1 milk and plant drink groups in any of the analyzed biochemical parameters relating to bone health, cardiometabolic status or immune function (*p* > 0.05). This indicates that all groups were comparable before the intervention, ensuring balanced conditions for assessing the effects of the beverages in subsequent stages of the study.

### 3.5. Dietary Assessment

#### 3.5.1. Milk/Plant Drink-Specific Food Frequency Questionnaire

Participants recorded daily intake of their assigned drink as well as the type and amount of milk and milk-containing products (e.g., coffee, tea, and hot chocolate) consumed outside the study, as well as other dairy products. This allowed for an assessment of habitual milk and dairy consumption outside the study.

#### 3.5.2. Food and Nutrient Intake

Food intake was assessed using a validated self-administered food frequency questionnaire (KomPAN) covering 22 foods consumed in the past year [25]. In addition, participants completed 3-day dietary records in the week preceding the baseline visit, weeks 4 and 8, and the week preceding the final visit (week 12). The three records included two weekdays and one weekend day. These were used to assess baseline nutrient intake and the impact of the intervention on nutrient intake. Data was also used to determine associations between diet and other study outcomes. Participants were given verbal and written instructions on how to measure and record their diet, and at visits, the research team reviewed their dietary records to ensure that adequate detail was provided and for any recording issues. To increase the reliability of portion size determination, during the interview at the follow-up visits, the 3-day record for each participant was verified by a dietician. Portion sizes were estimated using the standard Album of Photographs of Food Products and Dishes (National Institute of Public Health, NIH—National Research Institute). To ensure high data quality, reported consumption was verified by a qualified research team when the dietary records were returned. This procedure enabled any ambiguities regarding portion sizes or food descriptions to be clarified immediately. Data from the dietary records was entered into the Polish dietician software NUVERO NUVERO.PL by a nutrition student trained in dietary assessment and was used to estimate energy and nutrient intake. To ensure accuracy regarding the specific oat drink used in the study, a custom product entry was created in the database. The nutrient profile, particularly with regard to fortified nutrients such as calcium, vitamin D and vitamin B12, was manually entered based on the manufacturer’s declaration on the product label rather than relying on generic database entries.

To assess the validity of the reported energy intake (EI), we applied the Goldberg cut-off method, as updated by Black and Cole (2000) [26]. A key strength of our approach is that basal metabolic rate (BMR) was derived directly from DEXA analysis rather than estimated using predictive equations. Participants identified as underreporters based on these criteria were consequently excluded from further analyses.

## 4. Data Analysis

### 4.1. Sample Size Calculation

The sample size for each group was initially estimated at 40 volunteers (120 in total) using G*Power Software 3.1.9.7 [27]. These calculations were based on the assumption of small to medium effects (Cohen’s f = 0.25), a mixed ANOVA with repeated measures (three groups with two measurement points), a statistical power of 0.8, and an alpha significance level of 0.05. However, considering the challenges associated with drink consumption for 3 months and the planned measurements, it was decided to increase the size of all groups by 20%. Therefore, the final number of participants in each group was set at 50 (150 in total).

### 4.2. Statistical Analysis

Data analysis was performed using STATISTICA (version 13.1 PL, StatSoft Inc., Kraków, Poland). The normality of the variables was examined using the Shapiro–Wilk test and, when needed, appropriate transformations (log or Box-Cox) were applied where necessary. Continuous variables with a normal distribution were presented as means and standard deviations (SDs), with Student’s *t*-test or one-way analysis of variance being applied. For variables with a non-normal distribution, the Mann–Whitney U test was applied. Categorical variables were presented as numbers and percentages, and chi-squared (χ^2^) or Fisher’s exact tests were used. Associations between variables will be evaluated using linear regression when the dependent variable is a continuous variable and logistic regression when the dependent variable is categorical. Potential confounders will be taken from the multivariate analyses, only if they show a weak association (*p* < 0.20) with the dependent variables. The strength and direction of the correlation between two variables will be assessed using Pearson’s correlation test. A priori planned comparisons of within-group and between-group changes will focus on changes from baseline to the week 12 endpoint. The level of statistical significance was set at *p* ≤ 0.05 for all tests.

### 4.3. Data Management

#### 4.3.1. Privacy and Confidentiality

Identifying information was collected from participants at enrolment. Participants were assigned a unique code used on all study materials. The investigators responsible for data collection received training in Good Clinical Practice (GCP) to ensure the privacy and confidentiality of all study participants and the data collected. No identifying or identifiable information was disclosed in any reports or publications resulting from this study. All data was automatically anonymised before analysis.

#### 4.3.2. Data Storage

Paper data files were stored in locked filing cabinets in locked offices. Electronic data files were stored on a password-protected WULS server storage and were accessed and downloaded, as needed, to password-protected study team computers stored in locked facilities. The Principal Investigator will retain all study data for at least 15 years and participants may exercise their access rights at any time.

## 5. Expected Results and Contributions

The IMPA-CT Study is the first study in adults to identify and compare the effects of consumption of A2 milk, conventional milk and oat drinks on body composition, metabolic health and bone remodeling in the Polish population. Considering that the protocol was designed based on the best available evidence and previous experience, the IMPA-CT Study will contribute to the understanding of the therapeutic potential and safety profile or otherwise of β-casein A2/A2 milk and plant-based drinks.

The most well-documented beneficial effect of consuming A2 milk is its positive impact on the digestive system. The health-promoting properties of A2 milk mainly stem from the difference in casein structure and, consequently, the way it is digested. β-casein A2/A2 lacks cytomodulatory and immunosuppressive properties, meaning it does not cause negative gastrointestinal symptoms [2,14,28,29]. Furthermore, it is a source of bioactive peptides [3,30]. Milk containing A2/A2 casein may positively impact the morphology of the intestinal mucosa and modulate the intestinal microflora, thereby playing a key role in immune system function [31]. Milk containing β-casein A2/A2 can influence intestinal microbiota, increasing the production of short-chain fatty acids (SCFAs). These SCFAs play a vital role in maintaining intestinal health, particularly in regenerating and maintaining the function of intestinal epithelial cells. SCFAs such as isobutyrate are the main energy source for these cells and may therefore support gastrointestinal health. Furthermore, certain bacterial species, such as Bifidobacterium, may be more prevalent in individuals who consume A2 milk, potentially alleviating gastrointestinal symptoms [30,32]. Consumption of this milk has also been shown to increase the natural production of glutathione (GSH), an antioxidant that reduces the risk of diseases associated with oxidative stress [33,34]. However, the positive results obtained in experimental animal studies on the health effects of A2 milk on other metabolic or diet-related diseases have not always been confirmed in clinical studies involving humans [1,12,13,35].

In parallel, the IMPA-CT Study provided valuable insights into the health effects of plant-based beverages, which had rapidly gained popularity as alternatives to cow’s milk in recent years. These products, derived from a wide range of plant sources, including cereals, legumes, nuts, and seeds, differ markedly from cow’s milk in their nutritional value and bioavailability of key nutrients. While fortified plant-based beverages can offer comparable amounts of calcium and vitamin D to cow’s milk, they often have lower protein content and a distinct amino acid profile, with bioavailability further influenced by antinutritional factors such as phytates and oxalates. Moreover, plant-based drinks may contain bioactive compounds such as flavonoids, phytosterols, and beta-glucans, which could exert additional health effects, particularly cardiovascular and metabolic health [19,36]. However, the long-term impact of replacing dairy products with plant-based beverages remains insufficiently understood, with limited clinical data available beyond soy-based drinks [20,36,37]. Considering the increasing use of these beverages in varied age and dietary groups, including individuals with lactose intolerance, cow’s milk protein allergy, or those following plant-based diets, the IMPA-CT Study addressed a relevant knowledge gap by evaluating the effects of a selected fortified plant-based drink on body composition, bone metabolism, and metabolic parameters in adults from the Polish population. This comprehensive assessment will contribute to establishing evidence-based recommendations on the nutritional adequacy and safety profile of plant-based beverages as milk substitutes.

## Figures and Tables

**Table 1:** Basic nutritional values of tested drinks.

Nutritional Value	A2 Milk	A1 Milk	Oat Drink
Energy value, kcal	67	60	50
Fat, g	3.8	3.2	1.5
of which saturated acids, g	6.0	2.1	0.2
Carbohydrate, g	4.7	4.7	8.3
of which sugars, g	4.7	4.7	5.6
Protein, g	3.4	3.2	0.8
Salt, g	0.1	0.1	0.1
Calcium, mg	110	105	120
Riboflavin, mg	0.17	0.17	0.21
Vitamin B12, µg	0.25	0.25	0.38
Vitamin D, µg	0.03	0.03	0.75

**Table 2:** Baseline characteristics of the study groups before randomization.

Variables	A2 Milk Group (n = 54)	A1 Milk Group (n = 54)	Plant Drink Group (n = 54)	*p*-Value
Age	46.7 ± 8.1	46.0 ± 7.6	46.0 ± 7.4	0.926 *
Sex, %				
Woman	38	38	40	0.868 **
Man	16	16	14	
BMI, kg/m^2^	26.2 ± 4.9	25.7 ± 4.2	25.8 ± 4.8	0.918 *
%FM (DEXA)	35.5 ± 7.0	34.4 ± 7.2	33.5 ± 9.4	0.673 *
z-score (DEXA)	0.46 ± 0.96	0.17 ± 0.89	0.22 ± 0.92	0.402 *
Smoking, %				
Yes	4	5	5	0.925 **
No	48	47	47	
Alcohol, %				
Never or almost never	20	20	18	0.367 **
Less than once a week	18	12	11	
Once a week	11	10	15	
2–4 times a week	2	9	7	
5–6 times a week	1	1	0	

* Kruskal–Wallis test; ** chi^2^ test, *p* ≤ 0.05.

**Table 3:** Biochemical testing panel in IMPA-CT study.

Health Category	Material	Biomarker	Purpose/Rationale	Reference Values
**Bone health**	Blood	Calcium (Ca), mM/L	Marker of bone homeostasis—monitoring plasma calcium levels	2.15–2.50 mM/L (adults)
		1,25(OH)_2_D_3_ (Calcitriol), ng/mL	Assesses active vitamin D, regulating calcium metabolism and bone health	deficiency: 0–20 suboptimal level: 20–30 optimal level: 30–50 high level: 50–100 potentially toxic level: > 100
		P1NP (Procollagen type I *N*-terminal propeptide), ng/mL	Marker of bone formation—reflects osteoblast activity	men— premenopause women: 15–59 postmenopause women: 20–76
		ALP (alkaline phosphatase), U/L	Indicator of bone formation—marker of bone metabolic activity	men: 40–130 women: 35–105
		bone fraction as % of alkaline phosphatase		32.3–37.3
		CTX-1 (C-terminal telopeptide of type I collagen), ng/mL	Marker of bone resorption—evaluates degradation of type I collagen	men: 0.03–0.59 premenopause women: 0.039–0.575 postmenopause women: 0.105–1.017
	Urine	Calcium (Ca)	Marker of bone resorption—monitoring urinary calcium excretion	-
		Deoxypyridinoline, nM/mM creatinine	Marker of bone resorption—collagen breakdown product excreted in urine	men: 2.3–5.4 women: 3.0–7.4
		Creatinine, nM/L	Assesses renal function and normalizes other urinary biomarker results	m: 2.1–34.7 women: 1.3–24.6
**Cardiometabolic health**		Lipid profile (total cholesterol (TC), HDL, LDL, triglycerides (TGs)), mg/dL	Assesses general metabolic health and cardiovascular risk	TC: 115–190 (adults) HDL: men > 40; women > 45 LDL: <115 for people with low cardiovascular risk TG: <100 (adults)
		Glucose, mg/dL	Assessment of blood glucose control	70–99 (adults)
		Insulin, µIU/mL	Evaluation of glucose metabolism and insulin resistance	2.6–24.9 (adults)
	Blood	IGF-1 (insulin-like growth factor-1), ng/mL	Indicators of metabolic processes and growth hormone activity	men: 118–253 women: 60.7–182
		IGFBP-3 (IGF-binding protein (BP)-3), ng/mL		men: 3046–5955 women: 2512–5963
		hsCRP (high-sensitivity *C*-reactive protein), mg/L	Marker of inflammation and cardiovascular risk.	low cardiovascular risk < 1 moderate cardiovascular risk: 1–3 high cardiovascular risk > 3
		Uric acid, mg/dL	Assesses purine metabolism and risk of gout.	men: 3.4–7.0 women: 2.4–5.7
		Creatinine, mg/dL	Indicator of kidney function and metabolic balance	men: 0.7–1.2 women: 0.5–0.9
**Allergy/** **Immune health**	Blood	sIgE (allergen-specific Immunoglobulin E) kU/L	Marker for casein allergy diagnosis	<0.35 (negative)
		IgA (Immunoglobulin A), g/L	Indicators of immune system function	0.7–4.0
		IgG (Immunoglobulin G), g/L		7–16

- no value.

**Table 4:** Biochemical characteristics of the study participants after randomization.

Health Category	Material	Biomarker	A2 Milk Group (n = 54)	A1 Milk Group (n = 54)	Plant Drink Group (n = 54)	*p*-Value *
**Bone health**	Blood	Ca, mM/L	2.4 ± 0.1	2.4 ± 0.1	2.4 ± 0.1	0.854
		1,25(OH)_2_D_3_, ng/mL	35.2 ± 14.4	35.2 ± 12.8	34.6 ± 13.3	0.439
		P1NP, ng/mL	49.4 ± 29.2	45.5 ± 19.8	46.1 ± 21.0	0.958
		ALP, U/L	66.0 ± 17.9	71.5 ± 17.5	68.0 ± 19.6	0.387
		CTX-1, ng/mL	0.3 ± 0.2	0.3 ± 0.1	0.4 ± 0.4	0.993
	Urine	Ca, mmol/L	3.3 ± 2.0	2.8 ± 1.5	3.4 ± 2.0	0.337
		Deoxypyridinoline, nM DPD/mM creatinine	7.5 ± 13.1	7.5 ± 13.2	5.5 ± 1.7	0.888
		Creatinine	10.1 ± 5.5	10.9 ± 4.5	10.3 ± 5.4	0.289
**Cardiometabolic health**	Blood	Total cholesterol, mg/dL	202.2 ± 52.4	201.6 ± 37.9	198.3 ± 32.8	0.470
		HDL, mg/dL	66.1 ± 18.9	62.1 ± 15.2	67.6 ± 17.2	0.323
		LDL, mg/dL	118.9 ± 46.8	122.3 ± 36.4	115.9 ± 25.7	0.439
		Triglycerides, mg/dL	95.8 ± 58.5	94.4 ± 40.5	81.6 ± 40.8	0.436
		Glucose, mg/dL	88.3 ± 7.6	85.9 ± 6.6	86.0 ± 6.1	0.309
		Insulin, µIU/mL	7.3 ± 4.1	7.1 ± 4.0	7.1 ± 3.9	0.636
		IGF-1, ng/mL	141.1 ± 43.2	149.4 ± 51.2	144.6 ± 40.0	0.271
		IGFBP-3, ng/mL	4036 ± 795	4091 ± 1027	4153 ± 745	0.740
		hsCRP, mg/L	1.5 ± 1.8	1.7 ± 2.4	1.5 ± 2.8	0.299
		Uric acid, mg/dl	4.6 ± 1.0	4.6 ± 1.2	4.7 ± 1.1	0.993
		Creatinine, mg/dL	0.8 ± 0.1	0.9 ± 0.1	0.8 ± 0.2	0.826
**Allergy/** **Immune health**	Blood	sIgE, kU/L	<0.10 ^#^	<0.10 ^#^	<0.10 ^#^	0.923
		IgA, g/L	2.1 ± 0.7	2.3 ± 0.8	2.3 ± 1.1	0.785
		IgG, g/L	12.5 ± 2.1	12.4 ± 2.0	11.8 ± 1.9	0.271

* Kruskal–Wallis test; *p* ≤ 0.05. ^#^ sIgE: 2 individuals from the A1 milk group and 1 individual each from the A2 and plant-based milk groups had values > 0.10.

## Data Availability

The raw data supporting the conclusions of this article will be made available from the corresponding author upon reasonable request.
